# Systemic remodeling of the redox regulatory network due to RNAi perturbations of glutaredoxin 1, thioredoxin 1, and glucose-6-phosphate dehydrogenase

**DOI:** 10.1186/1752-0509-5-164

**Published:** 2011-10-13

**Authors:** Linda E Kippner, Nnenna A Finn, Shreya Shukla, Melissa L Kemp

**Affiliations:** 1 The Wallace H. Coulter Department of Biomedical Engineering, Georgia Institute of Technology and Emory University, Atlanta, GA, USA; 2 Department of Medicine, Division of Cardiology, Emory University, Atlanta, GA, USA; 3 Institute of Biomaterials and Biomedical Engineering, University of Toronto, Toronto, Ontario, Canada

## Abstract

**Background:**

Cellular clearance of reactive oxygen species is dependent on a network of tightly coupled redox enzymes; this network rapidly adapts to oxidative conditions such as aging, viral entry, or inflammation. Current widespread use of shRNA as a means to perturb specific redox couples may be misinterpreted if the targeted effects are not monitored in the context of potential global remodeling of the redox enzyme network.

**Results:**

Stable cell lines containing shRNA targets for glutaredoxin 1, thioredoxin 1, or glucose-6-phosphate dehydrogenase were generated in order to examine the changes in expression associated with altering cytosolic redox couples. A qRT PCR array revealed systemic off-target effects of altered antioxidant capacity and reactive oxygen species formation. Empty lentiviral particles generated numerous enzyme expression changes in comparison to uninfected cells, indicating an alteration in antioxidant capacity irrespective of a shRNA target. Of the three redox couples perturbed, glutaredoxin 1, attenuation produced the most numerous off-target effects with 10/28 genes assayed showing statistically significant changes. A multivariate analysis extracted strong co-variance between glutaredoxin 1 and peroxiredoxin 2 which was subsequently experimentally verified. Computational modeling of the peroxide clearance dynamics associated with the remodeling of the redox network indicated that the compromised antioxidant capacity compared across the knockdown cell lines was unequally affected by the changes in expression of off-target proteins.

**Conclusions:**

Our results suggest that targeted reduction of redox enzyme expression leads to widespread changes in off-target protein expression, changes that are well-insulated between sub-cellular compartments, but compensatory in both the production of and protection against intracellular reactive oxygen species. Our observations suggest that the use of lentivirus can in itself have off-target effects on dynamic responses to oxidative stress due to the changes in species concentrations.

## Background

The cellular redox environment is determined by numerous electron couples, including glutathione/glutathione disulfide (GSH/GSSG), reduced thioredoxin/oxidized thioredoxin, NAD(P)H/NAD(P)^+^, and cysteine/cystine, that transfer electrons during changes in intracellular oxidation state. These redox couples are in turn maintained out of equilibrium by a network of biochemical reactions, connected through a common set of substrates, products and co-factors. The overall behavior of this system is regarded as the antioxidant capacity of the cell and it determines the rate of reactive oxygen species (ROS) clearance from the cellular environment. In prior work, we have quantitatively described through computational modeling the collective properties of the redox enzymatic network as it pertains to exogenous hydrogen peroxide clearance from the cytosol and protein thiol oxidation/reduction [1]. This computational analysis demonstrated the relative contributions of peroxiredoxins, glutathione peroxidase, catalase, and protein thiol/disulfide oxidation to the removal of hydrogen peroxide from the intracellular environment. This model was specific to the Jurkat T-lymphocyte cell line, yet through the adjustment of initial enzyme, glutathione, and NAD(P)H concentrations this model could in principle simulate the oxidative protection mechanisms of other cell types.

Cellular variability in redox potentials is well-documented; for example, resting glutathione potential can range from -200 mV to -260 mV depending on cell type and culture conditions [2]. Furthermore, sensitivity of the cellular redox potential to cell cycle [3-5], viral load [6-10], and remodeling during inflammation [11,12] indicate that the set points of redox couples are readily altered by the "malleability" of redox enzyme gene expression. Numerous studies have examined the cDNA changes that occur across the genome in response to alterations in the oxidative environment (e.g. HIV infection, hypoxia, age); however these studies primarily report the significant up- or down-regulated gene hits from the conditions assayed without consideration of the subtle changes that could occur across the redox network.

Given that the expression levels of redox enzymes are readily altered, we asked whether targeted perturbation of specific redox couples would result in global remodeling of the redox enzyme network. RNA interference has become a common tool for biologists to quickly reduce protein levels in order to explore gene function with greater specificity than small molecule inhibitors can provide. Non-overlapping sequences of short hairpin RNA (shRNA) which have variable efficiency of interference can be exploited to generate "epi-allelic" cell lines with a range of protein silencing [13,14]. As RNAi is utilized with greater frequency to investigate the role of oxidative protein thiol modifications in cellular function [15-22], it is important to consider the specificity of RNAi perturbations with respect to intracellular oxidant sources and sinks. The introduction of viral particles induces oxidative stress and can alter cellular antioxidant levels; this alteration of cellular protein levels [23,24] may have unexpected redox-related consequences beyond potential off-target silencing, consequences that may substantially alter the redox capacity of the knockdown cells.

In order to discern changes in the redox environment that are primarily due to the on-target effects of viral incorporation as opposed to the off-target remodeling effects of redox couple perturbations, we performed a systematic analysis of the changes in expression of 28 redox-related genes due to stable RNAi knockdown of glutaredoxin 1 (Grx1), thioredoxin 1 (Trx1), or glucose-6-phosphate dehydrogenase (G6PD). We used computational programming to compare the redox ramifications of non-specific mRNA alterations to the redox ramifications of the idealized targeted knockdown scenarios. The dynamic analysis of intracellular hydrogen peroxide clearance indicates that surprisingly, while perturbing Trx1, Grx1, and G6PD have large consequences on antioxidant capacity, additional non-specific effects likely only modify the redox capacity of Grx1 shRNA transduced cells.

## Results

### Knockdown creation and validation

**Figure 1:**
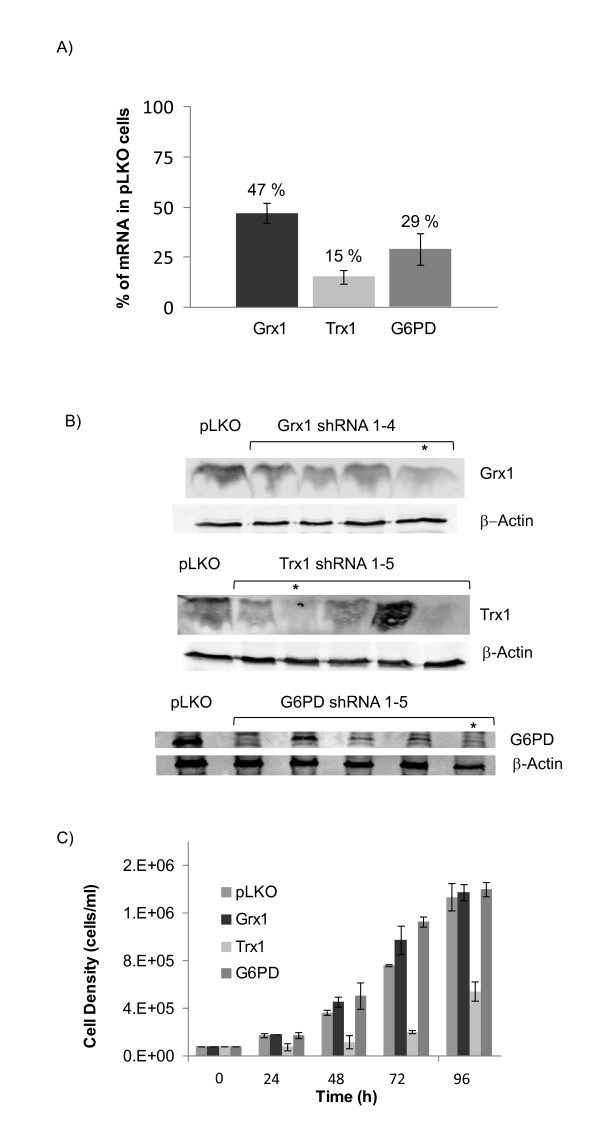
**Characterization of shRNA cell lines**. (A) β-Actin normalized mRNA levels of target silencing in selected cell lines with respect to pLKO. (B) Expression levels of target protein after stable shRNA silencing of Grx1, Trx1, and G6PD in Jurkat cells compared to pLKO empty vector control. Selected shRNA sequence is denoted with asterisk. (C) Growth curves of selected stable knockdown cell lines show changes in cell proliferation rates between shRNA and control cells. Error bars represent ± standard error (n = 3).

Lentiviral particles containing unique, non-overlapping mRNA sequences for each gene were used to generate shRNA-silenced Jurkat cell lines targeting Grx1, Trx1, and G6PD. Stable cell lines were created for each shRNA by puromycin selection. The most efficient shRNA for each gene of interest showed greater than 50% perturbation of mRNA levels as measured by qRT PCR (Figure 1A) and protein levels were further confirmed by western blot (Figure 1B). The fold changes in Grx1, Trx1, and G6PD levels were normalized relative to a housekeeping gene, β-actin, and compared to mRNA levels in the empty lentivirus (pLKO.1-puro) cells. The stable knockdown cell lines showed phenotypic differences in their growth rates (Figure 1C), with the Trx1 shRNA cells doubling at a slower pace than the other lines.

### Variability of non-target redox enzymes

**Figure 2:**
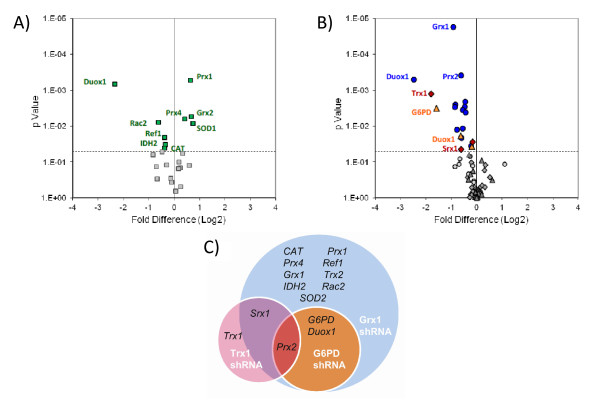
**Variability of non-target redox enzymes**. (A) Changes in off-target expression levels for wild-type Jurkat cells relative to pLKO empty vector control cells. Genes with significantly altered mRNA levels are colored green. (B) Changes in off-target expression levels for Grx1 shRNA (blue, circles), Trx1 shRNA (red, diamonds) and G6PD shRNA (orange, triangles), relative to pLKO control. (C) Venn diagram of significant gene changes. Statistical significance for all plots based upon p < 0.05.

We designed a PCR array consisting of 28 gene targets (Table 1) to examine the systematic variability of other non-targeted redox enzymes in the presence of shRNA lentiparticles. The genes selected for analysis were chosen to provide further insight into effects of lentiviral introduction on proteins that coordinate H_2_O_2 _production, transport, and clearance from the intracellular environment. We first compared wild-type Jurkat cells to cells that had been stably transduced with empty lentivirus (pLKO.1-puro) and observed significant changes in cellular mRNA levels (Figure 2A, Additional file 1). The largest statistically significant fold-changes were observed in Duox1, Prx1, Grx2, and SOD1 mRNA levels (p < 0.05). We attribute these large changes (> ± 50%) in the redox regulatory network to a response to the cellular stress caused by viral integration into the genome. Minor but statistically significant changes of ± 20-50% were observed in mRNA of Prx4, Rac2, Ref1, IDH2, and CAT. All further references to the knockdown cell lines will be with respect to the pLKO control cells unless explicitly stated.

**Table 1:** PCR array target list

Gene Symbol	Alias	Accession #	Official Full Name
CAT	MGC138422/MGC138424	NM_001752	Catalase

GPX1	GSHPX1/MGC14399/MGC88245	NM_000581	Glutathione peroxidase 1

GSR	MGC78522	NM_000637	Glutathione reductase

PRDX1	MSP23/NKEFA/PAG/PAGA/PAGB/PRX1/PRXI/TDPX2	NM_002574	Peroxiredoxin 1

PRDX2	MGC4104/NKEFB/PRP/PRX2/PRXII/TDPX1/TSA	NM_005809	Peroxiredoxin 2

PRDX4	AOE37-2/PRX-4	NM_006406	Peroxiredoxin 4

APEX1	APE/APE1/APEN/APEX/APX/HAP1/REF1	NM_080649	APEX nuclease (multifunctional DNA repair enzyme) 1

SRXN1	C20orf139/Npn3/SRX1/YKL086W/dJ850E9.2	NM_080725	Sulfiredoxin 1 homolog (S. cerevisiae)

TXNRD1	GRIM-12/MGC9145/TR/TR1/TRXR1/TXNR	NM_003330	Thioredoxin reductase 1

GLRX	GRX/GRX1/MGC117407	NM_002064	Glutaredoxin (thioltransferase)

GLRX2	GRX2/bA101E13.1	NM_197962	Glutaredoxin 2

TXN	DKFZp686B1993/MGC61975/TRX/TRX1	NM_003329	Thioredoxin

TXN2	MT-TRX/MTRX/TRX2	NM_012473	Thioredoxin 2

G6PD	G6PD1	NM_000402	Glucose-6-phosphate dehydrogenase

IDH1	IDCD/IDH/IDP/IDPC/PICD	NM_005896	Isocitrate dehydrogenase 1 (NADP+), soluble

IDH2	ICD-M/IDH/IDHM/IDP/IDPM/mNADP-IDH	NM_002168	Isocitrate dehydrogenase 2 (NADP+), mitochondrial

AQP8	-	NM_001169	Aquaporin 8

DUOX1	LNOX1/MGC138840/MGC138841/NOXEF1/THOX1	NM_175940	Dual oxidase 1

DUOX2	LNOX2/NOXEF2/P138TOX/THOX2	NM_014080	Dual oxidase 2

NOX1	GP91-2/MOX1/NOH-1/NOH1	NM_007052	NADPH oxidase 1

CYBB	CGD/GP91-1/GP91PHOX/GP91PHOX/NOX2/p91-PHOX	NM_000397	Cytochrome b-245, beta polypeptide

NOX3	GP91-3	NM_015718	NADPH oxidase 3

NOX4	KOX/KOX-1/RENOX	NM_016931	NADPH oxidase 4

NOX5	MGC149776/MGC149777/NOX5A/NOX5B	NM_024505	NADPH oxidase, EF-hand calcium binding domain 5

RAC1	MGC111543/MIG5/TC-25/p21-Rac1	NM_006908	Ras-related C3 botulinum toxin substrate 1 (rho family, small GTP binding protein Rac1)

RAC2	EN-7/Gx/HSPC022	NM_002872	Ras-related C3 botulinum toxin substrate 2 (rho family, small GTP binding protein Rac2)

SOD1	ALS/ALS1/IPOA/SOD/homodimer	NM_000454	Superoxide dismutase 1, soluble

SOD2	IPO-B/MNSOD/Mn-SOD	NM_000636	Superoxide dismutase 2, mitochondrial

ACTB	PS1TP5BP1	NM_001101	Actin, beta

The official full names, gene symbols, aliases, and accession numbers for the mRNA targets included in the PCR array.

The three silenced cell lines showed common non-specific effects as well as more target-specific variation as measured by qRT PCR. Surprisingly, silencing Trx1, Grx1, and G6PD each led to statistically significant decreases in Prx2 mRNA levels (11%, 35%, 13% expression, respectively). The Trx1 and Grx1 silenced cells also showed common reduction in sulfiredoxin 1 (Srx1) mRNA levels, while the Grx1- and G6PD-silenced cells both experienced reduced levels of Duox1 mRNA. The Grx1-silenced cells showed the most widespread off-target changes with respect to the control cells, with mRNA levels of 8 additional proteins affected: Prx4, IDH2, Ref1, Rac2, CAT, SOD2, G6PD, Trx2 (Figure 2B,C, Additional File 1).

In order to better determine what portion of the observed changes in mRNA levels was due to off-target effects of the shRNA and what portion could be attributed to lentiviral infection, we also compared the pLKO and shRNA cells to wild-type Jurkat cells (Additional File 1). With the exception of Prx2 for Trx1 and G6PD shRNA cells and Duox1 for G6PD shRNA cells, none of the significant changes in mRNA levels in our shRNA cells as compared to pLKO were accompanied by a similar change in mRNA levels in shRNA and pLKO cells when compared to wild-type cells.

**Figure 3:**
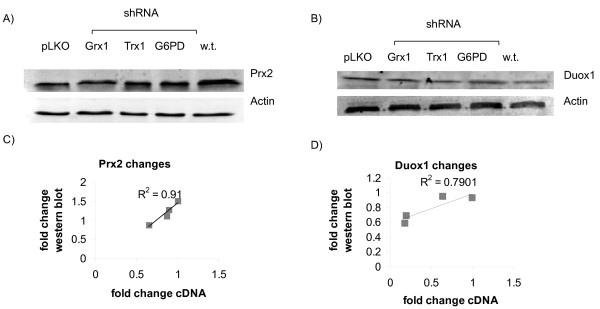
**Correlation between protein and mRNA levels**. Representative blots showing variation in (A) Prx2 and (B) Duox1 protein levels due to off-target effects of lentiviral shRNA. Correlation between protein levels derived from these blots and mRNA level for (C) Prx2 and (D) Duox1 in shRNA cell lines and wild type Jurkat cells.

In order to determine whether the measured changes in mRNA levels detected in our array reflected true changes in protein expression, each cell line was further assayed by western blot for Duox1 and Prx2 protein levels. These proteins could be further investigated because decreased levels of silencing were diverse enough in all three shRNA targets to create a broad range of values. As shown in Figure 3, the mRNA levels strongly correlated to protein levels for Duox1 (R^2 ^= 0.79), while the relation was stronger for Prx2 (R^2 ^= 0.91). Because the wild-type, uninfected cells represented an additional independent point to relate mRNA to protein, this measurement was included in the regression.

### Evaluation of co-variance among redox enzymes

**Figure 4:**
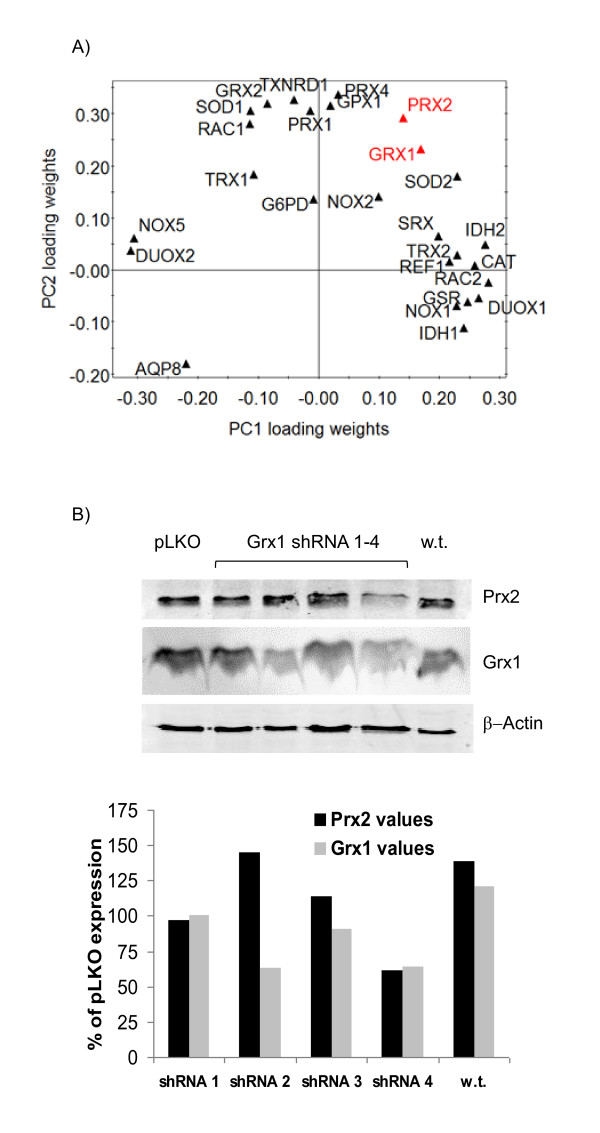
**Prediction of co-variance**. (A) Loadings plot of principal component analysis indicates strong co-variance between Grx1 and Prx2. (B) Off-target "modulation" of Prx2 by variable silencing of Grx1 in epi-allelic cell lines shows co-variance of Grx1 and Prx2 levels in three of four cell lines.

To evaluate patterns of co-variance among the mRNA levels, we modeled the array data by principal component analysis. This statistical modeling method, which collapses multivariate data into reduced dimensions, is useful for extracting patterns of co-variance across multiple observations. We created a data matrix consisting of five independent cell line observations: wild-type Jurkat cells, pLKO vector control, Grx1 shRNA, Trx1 shRNA and G6PD shRNA. Two principal components, or latent variables, were sufficient to explain 77.5% of the data variance, with PC1 capturing 44.4% and PC2 capturing 34.1%. The loadings plot as shown in Figure 4A illustrates relationships between genes. For example, the Trx2 and Ref1 mRNA levels are most tightly related among all variables as visualized by Euclidean distance. Some protein isoforms, such as the isocitrate dehydrogenases (IDH1, IDH2) map similarly in their contribution to the model. Likewise, the clustering of peroxiredoxin isoforms in the second principal component indicates that these proteins similarly contribute to the overall variance of the dataset, and likely reflect coordinated up- and down-regulation across the cell lines. Other isoform pairs (Trx1/Trx2 and SOD1/SOD2) show little co-variance and instead suggest insularity between mitochondrial and cytosol-specific proteins. Principal component analysis also highlights anticorrelative relationships by locations in opposing quadrants. In contrast to the IDH and Prx proteins, other isoforms have anticorrelative relationships, suggesting a compensatory effect in which cells up-regulate one gene to protect against the down-regulation of another. Protein pairs that fall within this category include Duox1/Duox2 and Nox1/Nox5.

Having established that our library of cell lines exhibited variation in both Prx2 mRNA and protein levels in response to the silencing of a variety of components within the redox regulatory network, we next asked if it was possible to modulate non-specific protein levels by shRNA-induced gradations in one of the redox couples. From the principal component analysis (Figure 4A), we predicted that Grx1 and Prx2 would strongly co-vary in levels. Three additional Grx1 shRNA stable cell lines ranging in efficiency from 20 to 65% knockdown levels were probed for Grx1 and Prx1, as was the wild-type cell line. We found that the Prx2 protein levels were co-expressed with Grx1 in three of our four cell lines (Figure 4B).

### Cellular responses to oxidative stress

Because our results suggested that the shRNA cells possess a reduced capacity to handle oxidative insult, we tested the functional consequences of a perturbed redox couple in the presence of a bolus addition of 100 μM hydrogen peroxide. Previously, a computational model of cellular hydrogen peroxide clearance was developed and optimized for wild-type Jurkat cells [1]. Built primarily using mass action kinetics, this model is the most comprehensive to date for examining the relative contributions of the peroxiredoxin system, protein S-thiolation, catalase, and glutathione/glutathione peroxidase mechanisms for removing hydrogen peroxide from the intracellular cytosolic environment. The model is accurate for up to one hour of dynamic simulation post-addition of an exogenous bolus of hydrogen peroxide ranging from 0-100 μM and is capable of explaining the independence in timescales of the thioredoxin and glutathione redox couples. To make the model compatible with the enzymatic changes associated with lentiviral entry, parameters and initial conditions associated with the genes that had statistically significant differences by mRNA changes (p < 0.05) between the pLKO cell lines and each of the other cell lines (Figure 2) were altered (Table 2) to generate cell line-specific models. These models did not take into account possible changes in glutathione and NADPH content, nor potential post-translational alterations to proteins that could result from an altered basal redox status. Furthermore, as the model assumes a well-mixed compartment, it is incapable of describing the enzymatic production of H_2_O_2 _that would occur due to NADPH oxidases.

**Table 2:** Cell-line specific parameter sets for computational modeling

Initial condition/rate constant	Jurkat	pLKO	Grx1 shRNA	Trx1 shRNA	G6PD shRNA
Grx1-SH (μM)	1.2	1.2	**0.62**	1.2	1.2

Trx1_ox _+ Trx1_red _(μM)	0.505	0.505	0.505	**0.144**	0.505

G6PD (M⋅s^-1^)	3.75e-4	3.75e-4	**2.55e-4**	3.75e-4	**1.24e-4**

Prx2-SH_2 _(μM)	19.2	19.2	**12.5**	**17.1**	**16.7**

Srx1 (s^-1^)	3e-3	3e-3	**1.74e-3**	**1.95e-3**	3e-3

CAT (μM)	0.9	**1.17**	**0.8**	0.9	0.9

Numbers in bold represent altered parameters from the wild-type Jurkat cell line.

**Figure 5:**
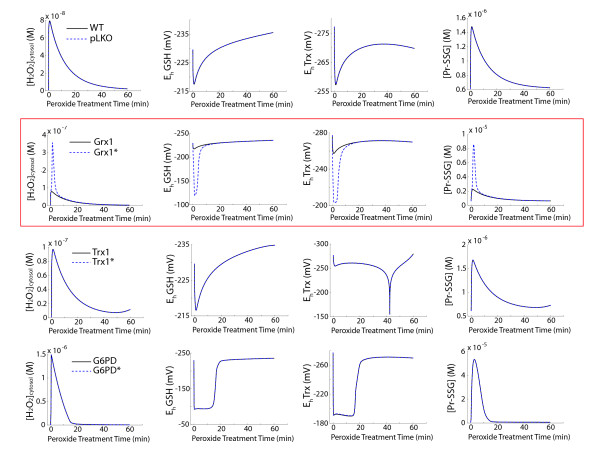
**Simulated consequences of the redox effects of off-target variations in shRNA perturbation**. Model predicted dynamics of intracellular H_2_O_2 _accumulation (first column), glutathione redox potential (second column), thioredoxin redox potential (third column), and Pr-SSG accumulation (fourth column) after a 100 μM extracellular H_2_O_2 _treatment. Solid lines represent the model-predicted behavior for the ideal KD cell lines with no off-target effects (w.t., Grx1, Trx1, G6PD), and dashed lines represent the model-predicted behavior for the experimentally characterized shRNA cell lines with qRT PCR-determined off-target effects (pLKO, Grx1*, Trx1*, G6PD*).

**Figure 6:**
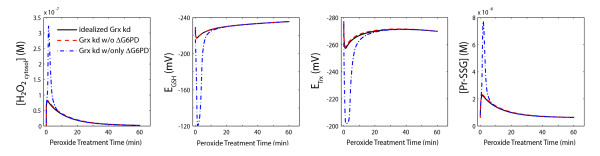
**G6PD off-target effect significantly alters intracellular redox capacity in knockdown cell lines**. Model predicted dynamics of intracellular H_2_O_2 _accumulation (first column), glutathione redox potential (second column), thioredoxin redox potential (third column), and Pr-SSG accumulation (fourth column) in the Grx1 knockdown cell line after a 100 μM extracellular H_2_O_2 _treatment. Solid black lines represent the model-predicted behavior for the ideal knockdown cell lines with no off-target effects (Grx1), red dashed lines represent the model-predicted behavior for the experimentally characterized shRNA cell lines with all qRT PCR-determined off-target effects except those affecting the G6PD enzyme, and blue dotted lines represent the model-predicted behavior for Grx1 silencing only in the presence of G6PD off-target effects.

We compared the simulations of these models in response to a 100 μM bolus treatment of hydrogen peroxide to the idealized case of perfect, specific targeting. These simulations were conducted to determine the effect of off-target alterations on the intracellular redox buffering capacity of the knockdown cell lines. Results of this analysis reveal that the off-target alterations caused by the introduction of pLKO shRNA, Trx1 shRNA, and G6PD shRNA lentiviral particles do not result in significant changes to the intracellular redox buffering capacity of the respective knockdown cell lines (Figure 5). However, the off-target alterations that result from Grx1 shRNA targeting result in significant alterations to the intracellular redox buffering capacity of the Grx1 knockdown cells (Figure 5). Upon further analysis of the off-target proteins that are affected by each of the shRNA constructs, it was discovered that the Grx1 shRNA lentiviral particle was the only lentiviral particle whose cellular introduction resulted in the off-target alteration of the G6PD enzyme. Based on this finding, we hypothesized that the off-target alteration of the G6PD enzyme was the primary additional modulation that was responsible for the significant differences seen between the ideal Grx1 knockdown behavior and the Grx1 knockdown behavior in the presence of off-target effects. To test this possibility, we simulated the hypothetical redox behavior of the Grx1 knockdown cell line without any off-target effects (i.e. idealized case) and with all off-target effects except for those involving the G6PD enzyme (Figure 6). These simulations suggest that if G6PD is not an off-target of Grx1 shRNA, then the Grx1 knockdown cells with off-target alterations should have the same redox buffering capacity in the presence of exogenous peroxide addition as the ideal Grx1 knockdown cells with no off-target alterations.

To establish the possible synergy of altered Grx1 and altered G6PD as drivers of redox state, we simulated conditions where only these two proteins are altered in the redox network versus all off-target effects except G6PD (Figure 6). Inclusion of the other Grx1 shRNA off-target proteins closely mimics the "idealized" case of just Grx1 silencing. In contrast, simulations of the Grx1 shRNA silencing solely in the presence of off-targeted reduction of G6PD dramatically influences the system behavior. This further indicates that the loss of robustness in redox capacity is due to a combined effect of Grx1 and G6PD.

## Discussion

Perturbations of cellular redox couples are instructive in applications that investigate the mechanisms of thiol-based signaling, protection from inflammatory reactive oxygen species and the metabolism of cysteines. Use of small molecule inhibitors such as carmustine (inhibitor of glutathione reductase) or dehydroepiandrosterone (inhibitor of G6PD) provide rapid alterations in the behavior of the redox enzymatic network, but this is potentially at the expense of specificity. Alternatively, small interfering RNA (siRNA) can transiently reduce protein levels with a high degree of specificity, but cellular characterization of the induced changes is often limited by the viability and transduction efficiency of the cells under investigation. Lentiviral infection of shRNA is purported to address both specificity and cell number limitations through the selection for stable incorporation of the interfering RNA within the genome. An additional benefit of stable silencing is the ability to assess long-term changes in the expression levels of other genes in response to the target. These changes take place on time-scales that vary among individual proteins; for example, in a genome-wide study of mouse embryonic stem cells, mRNA of NAD(P)H oxidase isoforms decay with half-lives on the order of 4-5 hours, while peroxiredoxins 1 & 2 and isocitrate dehydrogenase 1 & 2 had half-lives of 20-24 hours [25]. Consequently, when accounting for changes in mRNA and protein decay rates and sequential feedback, cellular remodeling in response to a silenced redox protein may not be fully implemented across all proteins to a new homeostatic state for several days. All of the shRNA cell lines used for this study had undergone at least three passages from the lentiviral infection and puromycin selection before characterization.

Because of the advantages to using shRNA, this method of examining redox regulation of cellular processes has been increasingly used to study effects of NAD(P)H supply, glutaredoxin, thioredoxin, and associated reductases [16-22]. Many of these studies rely on perturbation of the target protein in order to observe a phenotypic change, such as sensitivity to diamide-induced oxidative stress [19] or increased cellular senescence [22], and rely on the assumption that the rest of the antioxidant enzyme system remains intact. Our results, however, question this assumption by the changes observed in both the empty lentiviral construct and off-target changes due to specific protein silencing. We measured dramatic changes in redox protein mRNA levels due to the presence of the empty lentiviral vector, compared to uninfected cells. This is consistent with a vector-independent interferon response that is initiated by shRNA [26] or altered regulation of autocrine cytokines [27]. Furthermore, interferon γ stimulated response elements (ISRE) have been identified in the promoter regions of Nox1 and Nox2 [28,29] and therefore may potentially also control expression levels of other proteins involved in regulation of ROS, such as Duox1 and Rac1. Grx1 has been reported to be an essential regulator of interferon response factor-3 in Sendai virus infected cells [30]; if this role is conserved in all lentiviral infection, this mechanism may partially account for the sensitivity of the Jurkat cells to Grx1-specific RNA interference. Phosphorylation of the α subunit of eukaryotic initiation factor 2 (eIF2) through protein kinase R (PKR) is induced by IFN-γ and TNF-α [31,32] as well as a variety of stress conditions, including viral infection, and has been shown to down-regulate protein synthesis [33,34]. Therefore, lentiviral infection could potentially have post-translational effects on eIF2-α, thereby altering the regulation of protein synthesis. It should also be noted that treatment with aminoglycoside antibiotics has been shown to have effects on the expression levels of Grx1, and oxidative stress induced by antibiotics should be included as a potential cause for the changes we observed [35]. We have taken this into account in our experiments by using a control cell line (pLKO vector cells) that was cultured in the same puromycin-containing media as our shRNA cells. We compared our shRNA cells to this cell line and also compared mRNA levels in our shRNA and pLKO cell lines to that of wild-type cells (Figure 2, Additional file 1); however, a direct comparison of the effects of puromycin was not feasible, as puromycin was toxic to our wild-type cells.

In past cDNA microarray studies of responses associated with oxidative stress, transcriptional changes are reported as up- or down-regulated expression levels rather than the coordinated regulation of the redox-related enzymes [36-39]. Prior microarray studies on virally-infected T cells have not reported significant changes across the antioxidant enzyme network as observed in the present analysis, instead yielding one or two redox-related genes in the genomic arrays [40,41]. In contrast, other studies have demonstrated careful characterization of antioxidant enzyme changes, but only on a select few proteins (e.g. [42]). Systematic histological characterization of redox protein distribution provides complementary information, but does not allow for direct multivariate expression relationships on a large scale [43]. The intent of this study was to provide a more targeted approach with proteins that coordinate H_2_O_2 _production and clearance from the intracellular environment while still monitoring enough genes to observe systemic effects. Microarray and qRT PCR results may not always directly correspond to protein levels; however for a subset of proteins characterized in the present data (Grx1, Trx1, G6PD, Prx2, and Duox1) a linear relationship between mRNA and protein levels was observed.

Peroxiredoxins have emerged as important regulators of cellular redox status [1,44]. Among the coupled reactions of peroxiredoxin, thioredoxin, and sulfiredoxin, our network approach yielded novel insight into the regulation of this enzyme family (Figure 4A). A strong co-expression pattern emerged between the three peroxiredoxins measured (Prx1, 2, 4). Thioredoxin reductase also tightly co-varied with the peroxiredoxins while Trx1 showed less relation. A direct correlation between Srx1 and the rest of the peroxiredoxin branch was not established across the cell lines assayed, but the Trx1 knockdown cells showed a significant decrease in Srx1 (35%, p < 0.05). The off-target Srx1 compensation caused by attenuation of thioredoxin 1 expression may be more tightly regulated than our analysis suggests and could be explored further by epi-allelic strains of Trx1 shRNA targets. The distance between Prx2 and Grx1 in the principal component analysis demonstrated a stronger expression regulation between these two proteins than with Srx1 or Trx1. Because glutaredoxin 1 is not a substrate for the peroxiredoxins [45], this trend was unexpected but it was supported by analysis with additional cell lines. It has been postulated by Winterbourn and Hampton that additional functionality of peroxiredoxins through facilitated, indirect oxidation of other proteins could provide an alternate method of oxidative signal transmission [46]; if protein sulfenic acid is generated in this manner and subsequently protected by S-glutathionylation, than coordination between peroxiredoxins and Grx1 expression would be appropriate.

Disequilibrium of redox couples between the cytoplasm, ER, mitochondria, and nuclei has been elucidated through investigations of the differential subcellular sensitivities to oxidative stress [2,47-54]. The nature of the oxidative stress preferentially impacts different locations [55]; for example, EGF signaling induces Trx1 oxidation [51], while Trx2 is preferentially oxidized with TNF-α treatment [56]. The co-varying relationships and expression patterns illustrated in Figure 4A indicate that this insularity extends to a lack of up-regulated compensation of alternate isoforms when a cytosolic component (Trx1, Grx1) is altered, as we observed no statistical differences in the corresponding mitochondrial Trx2 and Grx2. In contrast, the computational modeling results suggest changes in the cytosolic NAD(P)H supply by G6PD silencing may result in enhanced mitochondrial NAD(P)H production by a dehydrogenase, IDH2. A significant decrease (35%, p < 0.05) in Trx2 upon Grx1 silencing was detected, however, but no known mechanism for communication between these compartmentalized redox proteins is known.

Changes due to the presence of the lentivirus could be accounted for with proper control experiments if not for the added non-specific off-target effects resulting from manipulating the various redox couples through RNA interference. The shRNA sequences used in the present study were for disparate protein targets with unique sequences, thus the changes observed are attributed to compensation by the cell for decreased reducing capabilities rather than direct silencing by RNA interference. Computational analysis allowed for the direct comparison of the idealized shRNA specificity to the more likely behavior if changes in mRNA levels reflect changes in protein translation. Although all the cell lines with lentivirus except the Grx1 shRNA knockdown cell line showed insignificant differences in antioxidant capacity due to off-target effects, it should be noted that not all the off-target effects detected by qRT PCR were capable of being simulated using the current cytosolic model. For example, downregulation of oxidative enzymes such as the Nox/Duox family, which would likely impact signaling-induced antioxidative responses, is not reflected in the models. This feature of ROS production is missing as the computational description only accounts for the dissipation of H_2_O_2 _by permeation through the cell membrane and clearance by glutathione, peroxisomes, and cytosolic enzymatic reactions. Our data shows that Duox1 was significantly downregulated in both the Grx1 and G6PD shRNA cell lines. It is possible that a more comprehensive model, one which is spatially descriptive and includes mitochondrial and membrane related antioxidant enzymes, could more readily show the resulting effect of off-target alterations on cellular antioxidant capacity. However, with the current modeling analysis, it is clear that off-target effects involving the G6PD enzyme system are more likely to result in significant changes in the overall redox capacity of the cell, compared to the idealized knockdown condition.

A caveat of our simulations is the numerous metabolic and post-translational modifications that are likely occurring in the cell lines. Although we maintained uniform redox state of NADPH/NADP^+^, Trx1-SH_2_/Trx1-SS, and GSH/GSSG for each cell-line specific model, each of these may be altered in response to the lentiviral constructs. For instance, G6PD knockdowns cells have been shown to exhibit an impaired ability to regenerate GSH [17]. Another caveat is that we have assumed that changes in mRNA levels will be reflected in protein levels. Although our detailed analysis of two proteins did show a linear correlation between mRNA and protein (Figure 3), this relation may not be upheld for all proteins due to translational regulation. Furthermore, proteins beyond the scope of our array involved in transport and metabolite synthesis may be altered to control the total available pools of NADPH and glutathione. Our analysis, therefore, is useful strictly as a means of examining one aspect of redox remodeling associated with viral infection and shRNA.

A surprising finding of our experimental and computational work is the dichotomy between the numerous changes that result at the mRNA level due to the introduction of lentiviral mRNA and the minor possible functional consequences in the overall ability of cells to handle acute, exogenous oxidative insult. Our shRNA perturbations of each redox couple indicated sensitivity to protein levels that were apparent only by inspection of multiple metrics of oxidative stress (Figure 5). With the exception of the Grx1/G6PD relationship, the off-target effects largely did not influence functionality of the redox enzymatic network as tested by our simulations. These results need to be investigated experimentally; for example, future studies of combined targeted silencing of both genes could characterize the necessary ratios of each protein required to maintain a robust redox system. Additional experiments are also necessary to establish whether the concomitant downregulation of ROS producing enzymes and enzymes involved in ROS scavenging is reflected in an increased resistance to cellular oxidation required for many signal transduction processes.

## Conclusions

In conclusion, this systemic study of gene co-regulation upholds findings of biochemical network analyses [1,57,58], namely that global cellular antioxidant capacity is a finely-tuned balance of multiple redox reactions tightly interconnected through common NAD(P)H supply, requiring systems-level characterization of reversible thiol oxidation. RNA interference provides a method to directly manipulate components of the pertinent oxidation and reduction enzymes where small molecule inhibitors are unavailable, but at a cost of unintentionally altering other aspects of the network. Attenuation of oxidases and associated regulatory components is one way that cells adjust to a perturbed antioxidant capacity. Computational approaches such as principal component analysis harness the unintended off-target effects in order to glean insight into compensatory measures that cells utilize as protection against oxidative damage; however our analysis points to challenges that can arise in implementing cell line-specific dynamic models if all non-specific effects are not built into the new parameter sets. While the application in the present study was RNAi-induced changes, this methodology of analysis would be useful in studying other forms of chronic reductive or oxidative stress.

## Methods

### Cell culture

Jurkat cells (ATCC, Manassas, VA) were cultured in RPMI-1640 medium (Sigma-Aldrich, St Louis, MO) supplemented with 10% FBS, 1% HEPES, 1% MEM non-essential amino acids, 1% sodium pyruvate, 100 U/ml penicillin, and 100 μg/ml streptomycin. Knockdown cells were cultured in the above medium with an addition of 4 μg/ml puromycin. Cells were cultured at a density of between 0.2 and 1.5 × 10^6 ^cells/ml in a humidified atmosphere of 5% CO_2 _and 95% air at 37°C. For proliferation studies, cell density was calculated by hemocytometer every 24 hours.

### RNA interference

Stable knockdowns were created from Jurkat cells by targeting the expression levels of Grx1, Trx1 or G6PD, using MISSION Lentiviral shRNA Transduction Particles (Sigma-Aldrich) according to manufacturer's protocol with puromycin selection (4 μg/ml). Jurkat cells with empty pLKO.1-puro lentiviral plasmid were used as control cells. MISSION TurboGFP™ Control Transduction Particles (Sigma-Aldrich) were used in a parallel experiment as a control for transduction efficiency.

### qRT PCR

RNA was isolated from cells using the RNeasy isolation kit (SABiosciences, Frederick, MD) with RNase-free DNase set (Qiagen, Valencia, CA) according to the manufacturer's protocol. 1 μg of RNA was used for reverse transcription. The reverse transcription reactions were performed using the SuperScript III First-Strand Synthesis System (Qiagen) for Trx1 shRNA validation and the RT2 first Strand Synthesis Kit (SABiosciences) for Grx1 shRNA validation and PCR array. Both kits were used according to manufacturer's protocol.

For shRNA validation, qRT PCR was run using primers for human Grx1, Trx1, and G6PD (SABiosciences). For detection of other mRNA levels, a custom RT2 Profiler PCR Array (SABiosciences) was used, according to manufacturer's protocol. A list of targets included in the array can be found in Table 1. PCR conditions were as follows: 10 min at 95°C; 40 cycles of 1 minute at 60°C and 15 seconds at 95°C; melt curve with ramp from 60°C to 95°C. All PCR reactions were run using the Step One Plus system (Applied Biosystems, Carlsbad, CA). Results were normalized to the expression of β-actin. Relative expression levels were calculated using the ΔCT method (2^-ΔCT^). All arrays were performed with triplicate sets of RNA isolation for each cell line for statistical analysis.

### Cell lysis for western blotting

For western blotting, 8 × 10^6 ^cells were lysed in 200 μl lysis buffer buffer containing 2% NP-40, 50 mM β-glycerophosphate, 10 mM NaP, 30 mM NaF, 50 mM Tris, pH 7.5, 150 mM NaCl, 1 mM benzamidine, 2 mM EGTA, 100 μM sodium orthovanadate, 1 mM DTT, 10 μg/ml aprotinin, 10 μg/ml leupeptin, 1 μg/ml pepstatin, 1 μg/ml microcystin-LR, and 1 mM PMSF. Cells were lysed on ice for one hour, followed by centrifugation for 10 minutes at 14000 rpm. Protein concentration was determined with the Micro BCA™ Assay Kit (ThermoFisher Scientific, Waltham, MA). For ΔPr-SSG blots, cells were lysed in a non- reducing buffer containing 2% NP-40, 50 mM Tris, pH 7.5, 150 mM NaCl, 1 mM benzamidine, 2 mM EGTA, 10 μg/ml aprotinin, 10 μg/ml leupeptin, 1 μg/ml pepstatin, 20 mM ethylmaleimide, and 1 mM PMSF.

### Western blotting

For western blots, 20 to 40 μg of total protein/sample was subjected to SDS-PAGE and transferred to PVDF membranes. For Pr-SSG blots, this was done under non-reducing conditions. The membrane was blocked with Near Infra-Red Blocking Buffer (Rockland Immunochemicals, Gilbertsville, PA) at room temperature for 1 hour. Primary antibodies were used at a dilution of 1:1000 in 10 ml blocking buffer and incubated over night at 4°C, followed by three washes in TBS-T. Secondary antibodies (IR dye 680CW donkey anti-mouse, IR dye 800CW anti-goat or IR dye 800CW donkey anti-rabbit, all from LI-COR Biosciences (Lincoln, NE), were all used at a dilution of 1:15000 in 10 ml blocking buffer and incubated for 1h at room temperature. This was followed by two washes in TBS-T and one wash in TBS. Imaging and image analysis were done using the Li-Cor Odyssey Infrared Imaging System with the Odyssey 2.1 software. β-actin was used as loading control. Primary antibody for Grx1 was purchased from R&D Systems (Minneapolis, MN), for full length Trx1 from BD Biosciences (Franklin Lakes, NJ), for Duox1 from Novus Biologicals (Littleton, CO), for Prx1 from Millipore, for Prx2 from Abcam (Cambridge, MA), for glutathione from Virogen (Watertown, MA), and for β-actin from Sigma-Aldrich.

### Statistical analysis

All values reported are the average of three independent biological replicates +/- standard error. Statistical significance is based upon the criteria of p < 0.05 for a Student's t-test (two-tailed, equal variance). Principal component analysis was performed by first mean centering and unit variance scaling the 2^ΔCt ^values (normalized to β-actin) to create a 5 × 28 data matrix. Simca-P software (Umetrics, Umeå, Sweden) provided stringency tests for determining the minimum number of latent variables (components) necessary to capture covariance in the data.

### Modeling simulations

A previously published model of the redox regulatory network was originally developed and optimized for wild-type Jurkat parameters [1]. This model was capable of simulating the clearance of 100 mM exogenous hydrogen peroxide from the cytosolic environment at a cell density of 10^6 ^cells/mL. A fundamental feature of the kinetic description is the use of changes in species from baseline instead of absolute concentrations due to the inherent disequilibrium in redox couples [2]. For the simulations presented here, all model parameters and files are identical to those in [1] except where noted below, and the kinetic equations can be found in Additional File 2. The model was adjusted to new baseline values for the pLKO, Grx1 shRNA, Trx1 shRNA, and G6PD shRNA by calculating the fold change for statistically significant differences (p < 0.05) in RNA levels of the following genes as described in Table 2: Prx2, G6PD, Grx1, Trx1, CAT, and Srx1. Changes to alternate isoforms of these genes (e.g Prx1, Prx4, Grx2, Trx2) were not factored into the model simulations in Figures 5 &6. The components that are represented in the model as species had their total concentrations adjusted accordingly. The components whose levels were implicitly reflected in a rate constant (G6PD, Srx1) had a fold-change alteration in parameter value. For the idealized cases, only the target of interest (Grx1, Trx1, and G6PD, respectively) was altered according to the altered mRNA value. Simulations of 60 minute responses were performed using Matlab 7.9.0 r2009b.

## List of abbreviations

G6PD: glucose-6-phosphate dehydrogenase; GSH: reduced glutathione; GSSG: glutathione disulfide; IDH: isocitrate dehydrogenase; IoM: ionomycin; Grx: glutaredoxin; NAD(P)H: nicotinamide adenine dinucleotide phosphate; RNAi: RNA interference; PMA: phorbol-12-myristate-13-acetate; Prx: peroxiredoxin; ROS: reactive oxygen species; shRNA: short interfering RNA; siRNA: small interfering RNA; Srx: sulfiredoxin 1; Trx: thioredoxin

## Authors' contributions

LEK participated in study design and coordination, created the shRNA cells lines, carried out all assays and statistical analyses, apart from computational modeling and principal component analysis, and drafted the manuscript. NAF contributed data for and performed the computational modeling, and participated in drafting the manuscript. SS participated in the creation of the shRNA cell lines. MLK conceived of the study, and participated in its design and coordination, performed the principal component analysis and drafted the manuscript. All authors read and approved the final manuscript.

## Supplementary Material

Additional file 1 **Significant changes in mRNA expression levels in shRNA and pLKO cells lines**. Significant changes (p < 0.05) in mRNA expression levels in shRNA cell lines and pLKO control cells compared to wild-type Jurkat cells, and in shRNA cells and wild-type cells compared to pLKO cells. Names in black represent targets that were expressed in all five of our cells lines, while names in red represent mRNA targets that were not expressed (Ct ≥ 35) in any of our cells lines. Click here for file

Additional file 2 **Description of computational model used for simulations in figures **5**and **6. Further details of model description and sources for parameter values can be found in Adimora NJ, Jones DP, Kemp ML. "A model of redox kinetics implicates the thiol proteome in cellular hydrogen peroxide responses." Antioxid Redox Signal 2010, 13:731-743. Each cell line represented in the simulations was modified by adjusting the subset of parameters listed in Table 2 of the main text. Click here for file

## References

[B1] Adimora NJ Jones DP Kemp ML A model of redox kinetics implicates the thiol proteome in cellular hydrogen peroxide responses Antioxid Redox Signal 2010 13 731 743 10.1089/ars.2009.2968 20121341 PMC2935341

[B2] Kemp M Go YM Jones DP Nonequilibrium thermodynamics of thiol/disulfide redox systems: a perspective on redox systems biology Free Radic Biol Med 2008 44 921 937 10.1016/j.freeradbiomed.2007.11.008 18155672 PMC2587159

[B3] Menon SG Goswami PC A redox cycle within the cell cycle: ring in the old with the new Oncogene 2006 26 1101 1109 16924237 10.1038/sj.onc.1209895

[B4] Menon SG Sarsour EH Spitz DR Higashikubo R Sturm M Zhang H Goswami PC Redox Regulation of the G1 to S Phase Transition in the Mouse Embryo Fibroblast Cell Cycle Cancer Research 2003 63 2109 2117 12727827

[B5] Conour JE Graham WV Gaskins HR A combined in vitro/bioinformatic investigation of redox regulatory mechanisms governing cell cycle progression Physiol Genomics 2004 18 196 205 10.1152/physiolgenomics.00058.2004 15138307

[B6] Casola A Burger N Liu T Jamaluddin M Brasier AR Garofalo RP Oxidant Tone Regulates RANTES Gene Expression in Airway Epithelial Cells Infected with Respiratory Syncytial Virus Journal of Biological Chemistry 2001 276 19715 19722 10.1074/jbc.M101526200 11259439

[B7] Machida K Cheng KTH Lai CK Jeng KS Sung VMH Lai MMC Hepatitis C Virus Triggers Mitochondrial Permeability Transition with Production of Reactive Oxygen Species, Leading to DNA Damage and STAT3 Activation The Journal of Virology 2006 80 7199 7207 10.1128/JVI.00321-06 PMC1489016 16809325

[B8] Elbim C Pillet S Prevost MH Preira A Girard PM Rogine N Matusani H Hakim J Israel N Gougerot-Pocidalo MA Redox and Activation Status of Monocytes from Human Immunodeficiency Virus-Infected Patients: Relationship with Viral Load The Journal of Virology 1999 73 4561 4566 10.1128/jvi.73.6.4561-4566.1999 PMC112496 10233914

[B9] Masutani H Ueda S Yodoi J The thioredoxin system in retroviral infection and apoptosis Cell Death Differ 2005 12 991 998 15818395 10.1038/sj.cdd.4401625

[B10] Davis DA Newcomb FM Starke DW Ott DE Mieyal JJ Yarchoan R Thioltransferase (Glutaredoxin) Is Detected Within HIV-1 and Can Regulate the Activity of Glutathionylated HIV-1 Protease in Vitro Journal of Biological Chemistry 1997 272 25935 25940 10.1074/jbc.272.41.25935 9325327

[B11] Reynaert NL Wouters EF Janssen-Heininger YM Modulation of glutaredoxin-1 expression in a mouse model of allergic airway disease Am J Respir Cell Mol Biol 2007 36 147 151 16980552 10.1165/rcmb.2006-0259RC PMC1899315

[B12] Maurice MM Nakamura H van der Voort EA van Vliet AI Staal FJ Tak PP Breedveld FC Verweij CL Evidence for the role of an altered redox state in hyporesponsiveness of synovial T cells in rheumatoid arthritis J Immunol 1997 158 1458 1465 9013992

[B13] Hemann MT Fridman JS Zilfou JT Hernando E Paddison PJ Cordon-Cardo C Hannon GJ Lowe SW An epi-allelic series of p53 hypomorphs created by stable RNAi produces distinct tumor phenotypes in vivo Nat Genet 2003 33 396 400 10.1038/ng1091 12567186

[B14] Wille L Kemp ML Sandy P Lewis CL Lauffenburger DA Epi-allelic Erk1 and Erk2 knockdown series for quantitative analysis of T cell Erk regulation and IL-2 production Mol Immunol 2007 44 3085 3091 10.1016/j.molimm.2007.02.008 17418417 PMC2692299

[B15] Lillig CH Lönn ME Enoksson M Fernandes AP Holmgren A Short interfering RNA-mediated silencing of glutaredoxin 2 increases the sensitivity of HeLa cells toward doxorubicin and phenylarsine oxide Proceedings of the National Academy of Sciences of the United States of America 2004 101 13227 13232 10.1073/pnas.0401896101 15328416 PMC516552

[B16] Saeed U Durgadoss L Valli RK Joshi DC Joshi PG Ravindranath V Knockdown of cytosolic glutaredoxin 1 leads to loss of mitochondrial membrane potential: implication in neurodegenerative diseases PLoS One 2008 3 e2459 10.1371/journal.pone.0002459 18560520 PMC2426930

[B17] Gao LP Cheng ML Chou HJ Yang YH Ho HY Chiu DT Ineffective GSH regeneration enhances G6PD-knockdown Hep G2 cell sensitivity to diamide-induced oxidative damage Free Radic Biol Med 2009 47 529 535 10.1016/j.freeradbiomed.2009.05.029 19497363

[B18] Ho YS Xiong Y Ho DS Gao J Chua BH Pai H Mieyal JJ Targeted disruption of the glutaredoxin 1 gene does not sensitize adult mice to tissue injury induced by ischemia/reperfusion and hyperoxia Free Radic Biol Med 2007 43 1299 1312 10.1016/j.freeradbiomed.2007.07.025 17893043 PMC2196211

[B19] Li D Zhu Y Tang Q Lu H Li H Yang Y Li Z Tong S A new G6PD knockdown tumor-cell line with reduced proliferation and increased susceptibility to oxidative stress Cancer Biother Radiopharm 2009 24 81 90 10.1089/cbr.2008.0494 19243250

[B20] Wei Q Jiang H Matthews CP Colburn NH Sulfiredoxin is an AP-1 target gene that is required for transformation and shows elevated expression in human skin malignancies Proc Natl Acad Sci USA 2008 105 19738 19743 10.1073/pnas.0810676105 19057013 PMC2604998

[B21] Wise TG Schafer DJ Lambeth LS Tyack SG Bruce MP Moore RJ Doran TJ Characterization and comparison of chicken U6 promoters for the expression of short hairpin RNAs Anim Biotechnol 2007 18 153 162 10.1080/10495390600867515 17612838

[B22] Young JJ Patel A Rai P Suppression of thioredoxin-1 induces premature senescence in normal human fibroblasts Biochem Biophys Res Commun 2010 392 363 368 10.1016/j.bbrc.2010.01.026 20074557

[B23] Yoshimura FK Luo X Zhao X Gerard HC Hudson AP Up-regulation of a cellular protein at the translational level by a retrovirus Proc Natl Acad Sci USA 2008 105 5543 5548 10.1073/pnas.0710526105 18378896 PMC2291115

[B24] Shenton D Smirnova JB Selley JN Carroll K Hubbard SJ Pavitt GD Ashe MP Grant CM Global translational responses to oxidative stress impact upon multiple levels of protein synthesis J Biol Chem 2006 281 29011 29021 10.1074/jbc.M601545200 16849329

[B25] Sharova LV Sharov AA Nedorezov T Piao Y Shaik N Ko MSH Database for mRNA Half-Life of 19 977 Genes Obtained by DNA Microarray Analysis of Pluripotent and Differentiating Mouse Embryonic Stem Cells DNA Research 2009 16 45 58 10.1093/dnares/dsn030 19001483 PMC2644350

[B26] Bridge AJ Pebernard S Ducraux A Nicoulaz AL Iggo R Induction of an interferon response by RNAi vectors in mammalian cells Nat Genet 2003 34 263 264 10.1038/ng1173 12796781

[B27] Schwarz KB Oxidative stress during viral infection: a review Free Radic Biol Med 1996 21 641 649 10.1016/0891-5849(96)00131-1 8891667

[B28] Lambeth JD Kawahara T Diebold B Regulation of Nox and Duox enzymatic activity and expression Free Radical Biology and Medicine 2007 43 319 331 10.1016/j.freeradbiomed.2007.03.028 17602947 PMC1989153

[B29] Kuwano Y Kawahara T Yamamoto H Teshima-Kondo S Tominaga K Masuda K Kishi K Morita K Rokutan K Interferon-gamma activates transcription of NADPH oxidase 1 gene and upregulates production of superoxide anion by human large intestinal epithelial cells Am J Physiol Cell Physiol 2006 290 C433 443 16162660 10.1152/ajpcell.00135.2005

[B30] Prinarakis E Chantzoura E Thanos D Spyrou G S-glutathionylation of IRF3 regulates IRF3-CBP interaction and activation of the IFN beta pathway EMBO J 2008 27 865 875 10.1038/emboj.2008.28 18309294 PMC2274937

[B31] Cheshire JL Williams BR Baldwin AS Jr Involvement of double-stranded RNA-activated protein kinase in the synergistic activation of nuclear factor-kappaB by tumor necrosis factor-alpha and gamma-interferon in preneuronal cells J Biol Chem 1999 274 4801 4806 10.1074/jbc.274.8.4801 9988719

[B32] Zamanian-Daryoush M Mogensen TH DiDonato JA Williams BR NF-kappaB activation by double-stranded-RNA-activated protein kinase (PKR) is mediated through NF-kappaB-inducing kinase and IkappaB kinase Mol Cell Biol 2000 20 1278 1290 10.1128/MCB.20.4.1278-1290.2000 10648614 PMC85265

[B33] Kaufman RJ Stress signaling from the lumen of the endoplasmic reticulum: coordination of gene transcriptional and translational controls Genes Dev 1999 13 1211 1233 10.1101/gad.13.10.1211 10346810

[B34] Sheikh MS Fornace AJ Jr Regulation of translation initiation following stress Oncogene 1999 18 6121 6128 10.1038/sj.onc.1203131 10557103

[B35] Hoppe G Chai YC Sears J Endogenous oxidoreductase expression is induced by aminoglycosides Arch Biochem Biophys 2003 414 19 23 10.1016/S0003-9861(03)00144-9 12745250

[B36] Go YM Park H Koval M Orr M Reed M Liang Y Smith D Pohl J Jones DP A key role for mitochondria in endothelial signaling by plasma cysteine/cystine redox potential Free Radic Biol Med 2010 48 275 283 10.1016/j.freeradbiomed.2009.10.050 19879942 PMC3057402

[B37] Herring TA Cuppett SL Zempleni J Genomic implications of H(2)O (2) for cell proliferation and growth of Caco-2 cells Dig Dis Sci 2007 52 3005 3015 10.1007/s10620-006-9663-6 17597414 PMC2136437

[B38] Spector A Li D Ma W Sun F Pavlidis P Differential amplification of gene expression in lens cell lines conditioned to survive peroxide stress Invest Ophthalmol Vis Sci 2002 43 3251 3264 12356832

[B39] Gorreta F Runfola TP VanMeter AJ Barzaghi D Chandhoke V Del Giacco L Identification of thioredoxin reductase 1-regulated genes using small interference RNA and cDNA microarray Cancer Biol Ther 2005 4 1079 1088 10.4161/cbt.4.10.1987 16096367

[B40] Ringrose JH Jeeninga RE Berkhout B Speijer D Proteomic studies reveal coordinated changes in T-cell expression patterns upon infection with human immunodeficiency virus type 1 J Virol 2008 82 4320 4330 10.1128/JVI.01819-07 18287243 PMC2293043

[B41] Chan EY Qian WJ Diamond DL Liu T Gritsenko MA Monroe ME Camp DG Smith RD Katze MG Quantitative analysis of human immunodeficiency virus type 1-infected CD4+ cell proteome: dysregulated cell cycle progression and nuclear transport coincide with robust virus production J Virol 2007 81 7571 7583 10.1128/JVI.00288-07 17494070 PMC1933372

[B42] Jacoby DB Choi AM Influenza virus induces expression of antioxidant genes in human epithelial cells Free Radic Biol Med 1994 16 821 824 10.1016/0891-5849(94)90198-8 8070686

[B43] Godoy JR Funke M Ackermann W Haunhorst P Oesteritz S Capani F Elsässer HP Lillig CH Redox atlas of the mouse: Immunohistochemical detection of glutaredoxin-, peroxiredoxin-, and thioredoxin-family proteins in various tissues of the laboratory mouse Biochimica et Biophysica Acta (BBA) - General Subjects in press Corrected Proof 10.1016/j.bbagen.2010.05.006 20682242

[B44] Low FM Hampton MB Peskin AV Winterbourn CC Peroxiredoxin 2 functions as a noncatalytic scavenger of low-level hydrogen peroxide in the erythrocyte Blood 2007 109 2611 2617 10.1182/blood-2006-09-048728 17105810

[B45] Chae HZ Kim HJ Kang SW Rhee SG Characterization of three isoforms of mammalian peroxiredoxin that reduce peroxides in the presence of thioredoxin Diabetes Res Clin Pract 1999 45 101 112 10.1016/S0168-8227(99)00037-6 10588361

[B46] Winterbourn CC Hampton MB Thiol chemistry and specificity in redox signaling Free Radic Biol Med 2008 45 549 561 10.1016/j.freeradbiomed.2008.05.004 18544350

[B47] Go YM Jones DP Redox compartmentalization in eukaryotic cells Biochim Biophys Acta 2008 1780 1273 1290 18267127 10.1016/j.bbagen.2008.01.011 PMC2601570

[B48] Go YM Pohl J Jones DP Quantification of redox conditions in the nucleus Methods Mol Biol 2009 464 303 317 10.1007/978-1-60327-461-6_17 18951192

[B49] Go YM Zeigler TR Johnson JM Gu L Hansen JM Jones DP Selective protection of nuclear thioredoxin-1 and glutathione redox systems against oxidation during glucose and glutamine deficiency in human colonic epithelial cells Free Radic Biol Med 2007 42 3 363 70 Epub 2006 Nov 10. 10.1016/j.freeradbiomed.2006.11.005 17210449 PMC1800831

[B50] Halvey PJ Hansen JM Johnson JM Go YM Samali A Jones DP Selective oxidative stress in cell nuclei by nuclear-targeted D-amino acid oxidase Antioxid Redox Signal 2007 9 807 816 10.1089/ars.2007.1526 17508907

[B51] Halvey PJ Watson WH Hansen JM Go YM Samali A Jones DP Compartmental oxidation of thiol-disulphide redox couples during epidermal growth factor signalling Biochem J 2005 386 215 219 10.1042/BJ20041829 15647005 PMC1134784

[B52] Jones DP Disruption of mitochondrial redox circuitry in oxidative stress Chem Biol Interact 2006 163 38 53 10.1016/j.cbi.2006.07.008 16970935

[B53] Watson WH Jones DP Oxidation of nuclear thioredoxin during oxidative stress FEBS Letters 2003 543 144 147 10.1016/S0014-5793(03)00430-7 12753922

[B54] Hansen JM Go YM Jones DP Nuclear and mitochondrial compartmentation of oxidative stress and redox signaling Annu Rev Pharmacol Toxicol 2006 46 215 234 10.1146/annurev.pharmtox.46.120604.141122 16402904

[B55] Cuddihy SL Winterbourn CC Hampton MB Assessment of redox changes to hydrogen peroxide-sensitive proteins during EGF signaling Antioxid Redox Signal 2011 15 167 174 10.1089/ars.2010.3843 21254838

[B56] Hansen JM Zhang H Jones DP Mitochondrial Thioredoxin-2 Has a Key Role in Determining Tumor Necrosis Factor-{alpha}-Induced Reactive Oxygen Species Generation, NF-{kappa}B Activation, and Apoptosis Toxicological Sciences 2006 91 643 650 10.1093/toxsci/kfj175 16574777

[B57] Ng CF Schafer FQ Buettner GR Rodgers VG The rate of cellular hydrogen peroxide removal shows dependency on GSH: mathematical insight into in vivo H2O2 and GPx concentrations Free Radic Res 2007 41 1201 1211 10.1080/10715760701625075 17886026 PMC2268624

[B58] Schafer FQ Buettner GR Redox environment of the cell as viewed through the redox state of the glutathione disulfide/glutathione couple Free Radic Biol Med 2001 30 1191 1212 10.1016/S0891-5849(01)00480-4 11368918

