# Supplementary material for: Systemic remodeling of the redox regulatory network due to RNAi perturbations of glutaredoxin 1, thioredoxin 1, and glucose-6-phosphate dehydrogenase
Source: BMC Syst Biol. 2011 Oct 13;5:164. doi: 10.1186/1752-0509-5-164 (PMC3199260; doi:10.1186/1752-0509-5-164)
Supplement: Additional file 1 — Significant changes in mRNA expression levels in shRNA and pLKO cells lines. Significant changes (p < 0.05) in mRNA expression levels in shRNA cell lines and pLKO control cells compared to wild-type Jurkat cells, and in shRNA cells and wild-type cells compared to pLKO cells. Names in black represent targets that were expressed in all five of our cells lines, while names in red represent mRNA targets that were not expressed (Ct ≥ 35) in any of our cells lines. [file 1752-0509-5-164-S1.PDF]

	Changes compared to wild-type									Changes compared to pLKO						
	Trx1 shRNA		G6PD shRNA		Grx1 shRNA		pLKO			Trx1 shRNA		G6PD shRNA		Grx1 shRNA		w.t.
**Gene Symbol**	**Log2(FC)**	**p Value**	**Log2(FC)**	**p Value**	**Log2(FC)**	**p Value**	**Log2(FC)**	**p Value**	**Gene Symbol**	**Log2(FC)**	**p Value**	**Log2(FC)**	**p Value**	**Log2(FC)**	**p Value**	**Log2(FC)**
CAT			0.444097201	0.023091979	-1.257712046	0.000983546	0.386443774	0.041358057	CAT					-0.561615626	0.003558369	-0.386443774
GPX1					-1.431850433	0.00103646			GPX1							
GSR									GSR							
PRDX1	-0.319551129	0.025626635	-0.633456231	0.006342395	-1.960039775	0.000356142	-0.643682798	0.00053749	PRDX1					-0.233816783	0.036223713	0.643682798
PRDX2					-1.74936676	5.84315E-05			PRDX2	-0.1626774	0.0277069	-0.195821126	0.036637074	-0.614979426	0.000389564	
PRDX4	-0.429704963	0.00137241	-0.42681853	0.041205272	-1.95269839	0.000177952	-0.414676348	0.006335573	PRDX4					-0.455481847	0.002081694	0.414676348
APEX1					-1.184022268	0.003632724	0.373452187	0.020380684	APEX1					-0.47493426	0.002845608	-0.373452187
SRXN1					-1.383929571	0.000466325			SRXN1	-0.6246443	0.0438786					
TXNRD1					-2.006031036	0.025687792			TXNRD1							
GLRX					-2.354386012	0.004228108			GLRX					-0.935614904	1.74131E-05	
GLRX2	-0.585129081	0.0109731	-0.730017665	0.002984928	-1.876590729	0.000223881	-0.674129168	0.005439961	GLRX2							0.674129168
TXN	-1.961231846	0.003534751			-1.525462786	0.003310809			TXN	-1.8113858	0.0012555					
TXN2					-1.385533015	0.015217843			TXN2					-0.607021332	0.021386216	
G6PD			-1.462068242	0.002441014	-1.49723498	0.002959574			G6PD			-1.597409566	0.003214057	-0.550036112	0.011471815	
IDH1	1.370188098	0.01391249							IDH1							
IDH2	0.482473712	0.089380136			-1.587453842	0.005694317	0.345824877	0.032086868	IDH2					-0.850738525	0.002539775	-0.345824877
AQP8									AQP8							
DUOX1	2.16922476	0.001517917	1.703991256	0.007574579			2.347567558	0.000666391	DUOX1			-0.643576304	0.01881708	-2.489041011	0.000499238	-2.347567558
DUOX2									DUOX2							
NOX1									NOX1							
CYBB									CYBB							
NOX3									NOX3							
NOX4									NOX4							
NOX5									NOX5							
RAC1			-0.539853732	0.00531766	-1.433111827	0.011035792			RAC1							
RAC2	0.722553592	0.004247462	0.557637848	0.006498566	-1.33496666	0.005201047	0.618503888	0.007873064	RAC2					-0.870930354	0.002953091	-0.618503888
SOD1	-0.613074599	0.001945752	-0.718765259	0.00411683	-1.804046949	0.001216395	-0.736918132	0.008477113	SOD1							0.736918132
SOD2					-1.412471135	0.002954379			SOD2					-0.442469915	0.004187063	
