# Supplementary material for: Systemic remodeling of the redox regulatory network due to RNAi perturbations of glutaredoxin 1, thioredoxin 1, and glucose-6-phosphate dehydrogenase
Source: BMC Syst Biol. 2011 Oct 13;5:164. doi: 10.1186/1752-0509-5-164 (PMC3199260; doi:10.1186/1752-0509-5-164)
Supplement: Additional file 2 — Description of computational model used for simulations in figures 5and 6. Further details of model description and sources for parameter values can be found in Adimora NJ, Jones DP, Kemp ML. "A model of redox kinetics implicates the thiol proteome in cellular hydrogen peroxide responses." Antioxid Redox Signal 2010, 13:731-743. Each cell line represented in the simulations was modified by adjusting the subset of parameters listed in Table 2 of the main text. [file 1752-0509-5-164-S2.DOCX]

**Supplemental Information**

**Description of computational model used for simulations in figures 5 and 6:**

Further details of model description and sources for parameter values can be found in Adimora NJ, Jones DP, Kemp ML. “A model of redox kinetics implicates the thiol proteome in cellular hydrogen peroxide responses.” *Antioxid Redox Signal* 2010, 13:731-743. Each cell line represented in the simulations was modified by adjusting the subset of parameters listed in Table 2 of the main text.

| **Description** | **Parameter** |
| --- | --- |
| H_2_O_2_ diffusion across plasma membrane | *Area_cell_ =*1.0 x 10^-5^ cm^2^ |
|  | *k_1_* = 1.0 x 10^-5^ cm s^-1^ |
| H_2_O_2_ basal intracellular generation | *k_2_* = 1.1 x 10^-7^ M s^-1^ |
|  | *Vol_cell_ =* 9.13 x 10^-13^ L |
| GPx_red_ oxidation | *k_3_* = 2.1 x 10^7^ M^-1^ s^-1^ |
| GPx_ox_ S-glutathionylation | *k_4_* = 4 x 10^4^ M^-1^ s^-1^ |
| GPx-SSG reduction by GSH | *k_5_* = 1 x 10^7^ M^-1^ s^-1^ |
| Peroxisomal catalase reaction | *k_6_* = 3.4 x 10^7^ M^-1^ s^-1^ |
|  | *CAT =* 9 x 10^-7^ M |
| K_m_ of NADP for G6PD reaction | *k_7_ =* 5.7 x 10^-5^ M |
| Prx-SH_2_ oxidation | *k_8_* = 4 x 10^7^ M^-1^ s^-1^ |
| Prx-SOH oxidation | *k_9_* = 7.2 x 10^4^ M^-1^ s^-1^ |
| Sulfiredoxin reaction | *k_10_* = 3 x 10^-3^ s^-1^ |
| Self-catalyzed disulfide formation of Prx-SS from Prx-SOH | *k_11_* = 15 s^-1^ |
| Reduction of Prx-SS by Trx1 | *k_12_* = 2.1 x 10^6^ M^-1^ s^-1^ |
| Auto-oxidation of GSH | *k_13_* = 7.4 x 10^-5^ s^-1^ |
| Protein monothiol oxidation | *k_14_* = 1 x 10^4^ M^-1^ s^-1^ |
| Protein S-glutathionylation from sulfenic acid | *k_15_* = 1.2 x 10^5^ M^-1^ s^-1^ |
| De-glutathionylation of Protein-SSG by Grx1 | *k_16_* = 9.1 x 10^4^ M^-1^ s^-1^ |
| De-glutathionylation of Grx1-SSG by GSH | *k_17_* = 3.7 x 10^4^ M^-1^ s^-1^ |
| Protein dithiol oxidation | *k_18_* = 5 x 10^5^ M^-1^ s^-1^ |
| Reduction of protein disulfide by Trx1 | *k_19_* = 1 x 10^5^ M^-1^ s^-1^ |
| Glutathione reductase reaction | *k_20_* = 3.2 x 10^6^ M^-1^ s^-1^ |
| Thioredoxin reductase reaction | *k_21_* = 2 x 10^7^ M^-1^ s^-1^ |
| V_max_ of NADP for G6PD reaction | *k_22_* = 3.75 x 10^-4^ M s^-1^ |
| Diffusion of H_2_O_2_ across peroxisomal membrane | *k_23_* = 3 x 10^-3^ cm s^-1^ |
|  | *Area_perox_ =* 3 x 10^-9^ cm^2^ |
|  | *Vol_perox_ =* 1.5 x 10^-14^ L |
| GSH synthesis | *k_24_* = 4.1 x 10^-7^ M s^-1^ |
| GSSG efflux | *k_25_* = 1.2 x 10^-8^ M s^-1^ |
| GSH + GSSG efflux | *k_26_* = 1.2 x 10^-7^ M s^-1^ |
| Trx1 efflux | *k_27_* = 7.5 x 10^-10^ M s^-1^ |
| Trx1 synthesis | *k_28_* = 7.0 x 10^-10^ M s^-1^ |

| **Species** | **Initial Condition (M)** |
| --- | --- |
| [H_2_O_2_]_media_ | 1 x 10^-4^ |
| [H_2_O_2_]_cyto_ | 1 x 10^-9^ |
| [GPx_red_] | 5 x 10^-5^ |
| [GPx_ox_] | 1 x 10^-14^ |
| [GPx-SSG] | 1 x 10^-14^ |
| [GSH] | 3.68 x 10^-4^ |
| [GSSG] | 1.78 x 10^-6^ |
| [Catalase] | 9 x 10^-7^ |
| [H_2_O_2_]_perox_ | 1 x 10^-10^ |
| [Prx2-(SH)_2_] | 1.92 x 10^-5^ |
| [Prx2-(SOH)] | 1 x 10^-14^ |
| [Prx2-(SOOH)] | 1 x 10^-14^ |
| [Prx2-SS] | 9.6 x 10^-8^ |
| [Trx1*_red_*] | 4.3 x 10^-7^ |
| [Trx1*_ox_*] | 7.5 x 10^-8^ |
| [Pr-SH]] | 1.22 x 10^-4^ |
| [Pr-SOH] | 6.1 x 10^-7^ |
| [Pr-SSG] | 6.1 x 10^-7^ |
| [Grx1-SH] | 1.2 x 10^-6^ |
| [Grx1-SSG] | 6.0 x 10^-9^ |
| [Pr-(SH)_2_] | 1.0 x 10^-3^ |
| [Pr-(SS)] | 5.5 x 10^-6^ |
| [NADPH] | 3.0 x 10^-5^ |
| [NADP^+^] | 3.0 x 10^-7^ |

$$\begin{matrix} \frac{dH_{2}O_{2media}}{dt}=k_{1}(H_{2}O_{2cyto}-H_{2}O_{2media})\cdot10^{-3}L/cm^{3}\cdot Area_{cell}\cdot10^{9}cells/L \\ \\ \frac{dH_{2}O_{2cyto}}{dt}=-k_{1}(H_{2}O_{2cyto}-H_{2}O_{2media})\cdot10^{-3}L/cm^{3}\cdot Area_{cell}/Vol_{cell}-2\cdot k_{23}\cdot H_{2}O_{2cyto}\cdot10^{-3}L/cm^{3}\cdot Area_{perox}/Vol_{perox}+ \\ k_{2}-[k_{3}GPx_{red}+k_{6}Cat+k_{8}Prx2_{SH}+k_{9}Prx2_{SOH}+k_{14}Pr_{SH}-k_{18}Pr_{SH2}](H_{2}O_{2cyto}-H_{2}O_{2cytoi}) \end{matrix}$$

$$\begin{matrix} \frac{dGPx_{red}}{dt}=-k_{3}GPx_{red}(H_{2}O_{2cyto}-H_{2}O_{2cytoi})+k_{5}GSH\cdot(GPx_{SSG}-GPx_{SSGi}) \\ \\ \frac{dGPx_{ox}}{dt}=k_{3}GPx_{red}(H_{2}O_{2cyto}-H_{2}O_{2cytoi})-k_{4}GSH\cdot(GPx_{ox}-GPx_{oxi}) \\ \\ \frac{dGPx_{SG}}{dt}=k_{4}GSH\cdot(GPx_{ox}-GPx_{oxi})-k_{5}GSH\cdot(GPx_{SSG}-GPx_{SSGi}) \end{matrix}$$

$$\begin{matrix} \frac{dGSH}{dt}=-k_{4}GSH\cdot(GPx_{ox}-GPx_{oxi})-k_{5}GSH\cdot(GPx_{SSG}-GPx_{SSGi})-2\cdot k_{13}GSH-k_{15}GSH\cdot(Pr_{SOH}-Pr_{SOHi}) \\ -k_{17}GSH\cdot(Grx1_{SSG}-Grx1_{SSGi})+2\cdot k_{20}NADPH\cdot(GSSG-GSSG_{i})+k_{24}-k_{26} \end{matrix}$$

$$\frac{dGSSG}{dt}=k_{5}GSH\cdot(GPx_{SSG}-GPx_{SSGi})+k_{13}GSH+k_{17}GSH\cdot(Grx1_{SSG}-Grx1_{SSGi})-k_{20}NADPH\cdot(GSSG-GSSG_{i})-k_{25}-k_{26}$$

$$\frac{dH_{2}O_{2perox}}{dt}=k_{23}H_{2}O_{2cyto}\cdot10^{-3}L/cm^{3}\cdot Area_{perox}/Vol_{perox}-k_{6}Cat\cdot(H_{2}O_{2perox}-H_{2}O_{2peroxi})$$

$$\begin{matrix} \frac{dPrx2_{SH2}}{dt}=-k_{8}Prx2_{SH2}\cdot(H_{2}O_{2cyto}-H_{2}O_{2cytoi})+k_{12}Trx1_{red}\cdot(Prx2_{SS}-Prx2_{SSi}) \\ \\ \frac{dPrx2_{SOH}}{dt}=(k_{8}Prx2_{SH2}-k_{9}Prx2_{SOH})(H_{2}O_{2\mathrm{int}}-H_{2}O_{2\mathrm{int}i})+k_{10}(Prx2_{SO2H}-Prx2_{SO2Hi})-k_{11}Prx2_{SOH} \\ \\ \frac{dPrx2_{SO2H}}{dt}=k_{9}Prx2_{SOH}\cdot(H_{2}O_{2cyto}-H_{2}O_{2cytoi})-k_{10}(Prx2_{SO2H}-Prx2_{SO2Hi}) \\ \\ \frac{dPrx2_{SS}}{dt}=k_{11}Prx2_{SOH}-k_{12}Trx1_{red}\cdot(Prx2_{SS}-Prx2_{SSi}) \end{matrix}$$

$$\begin{matrix} \frac{dTrx1_{red}}{dt}=-k_{12}Trx1_{red}\cdot(Prx2_{SS}-Prx2_{SSi})-k_{19}Trx1_{red}\cdot(\Pr_{SS}-\Pr_{SSi})+k_{21}NADPH\cdot(Trx1_{ox}-Trx1_{oxi})-k_{27}+k_{28} \\ \\ \frac{dTrx1_{ox}}{dt}=k_{12}Trx1_{red}\cdot(Prx2_{SS}-Prx2_{SSi})+k_{19}Trx1_{red}\cdot(\Pr_{SS}-\Pr_{SSi})-k_{21}NADPH\cdot(Trx1_{ox}-Trx1_{oxi}) \end{matrix}$$

$$\begin{matrix} \frac{dPr_{SH}}{dt}=-k_{14}Pr_{SH}\cdot(H_{2}O_{2cyto}-H_{2}O_{2cytoi})+k_{16}Grx1_{SH}(Pr_{SSG}-Pr_{SSGi}) \\ \\ \frac{dPr_{SOH}}{dt}=k_{14}Pr_{SH}\cdot(H_{2}O_{2cyto}-H_{2}O_{2cytoi})-k_{15}GSH\cdot(Pr_{SOH}-Pr_{SOHi}) \\ \\ \frac{dPr_{SSG}}{dt}=k_{15}GSH\cdot(Pr_{SOH}-Pr_{SOHi})-k_{16}Grx1_{SH}(Pr_{SSG}-Pr_{SSGi}) \end{matrix}$$

$$\begin{matrix} \frac{dGrx1_{SH}}{dt}=k_{17}GSH\cdot(Grx1_{SSG}-Grx1_{SSGi})-k_{16}Grx1_{SH}\cdot(Pr_{SSG}-Pr_{SSGi}) \\ \\ \frac{dGrx1_{SSG}}{dt}=k_{16}Grx1_{SH}\cdot(Pr_{SSG}-Pr_{SSGi})-k_{17}GSH\cdot(Grx1_{SSG}-Grx1_{SSGi}) \end{matrix}$$

$$\begin{matrix} \frac{dPr_{SH2}}{dt}=-k_{18}Pr_{SH2}\cdot(H_{2}O_{2cyto}-H_{2}O_{2cytoi})+k_{19}Trx1_{red}\cdot(Pr_{SS}-Pr_{SSi}) \\ \\ \frac{dPr_{SS}}{dt}=k_{18}Pr_{SH2}\cdot(H_{2}O_{2cyto}-H_{2}O_{2cytoi})-k_{19}Trx1_{red}\cdot(Pr_{SS}-Pr_{SSi}) \end{matrix}$$

$$\begin{matrix} \frac{dNADPH}{dt}=-k_{20}NADPH\cdot(GSSG-GSSG_{i})-k_{21}NADPH\cdot(Trx1_{ox}-Trx1_{oxi})+k_{22}(NADP^{+}-NADP^{+}_{i})/(k_{7}+NADP^{+}) \\ \\ \frac{dNADP^{+}}{dt}=k_{20}NADPH\cdot(GSSG-GSSG_{i})+k_{21}NADPH\cdot(Trx1_{ox}-Trx1_{oxi})-k_{22}(NADP^{+}-NADP^{+}_{i})/(k_{7}+NADP^{+}) \end{matrix}$$
